# External cervical resorption: diagnostic and treatment tips

**DOI:** 10.1590/2177-6709.21.5.019-025.oin

**Published:** 2016

**Authors:** Alberto Consolaro

**Affiliations:** 1 Full professor, Universidade de São Paulo (USP), School of Dentistry, Department of Orthodontics, Bauru, São Paulo, Brazil. Professor, Universidade de São Paulo (USP), School of Dentistry, Graduate Program, Ribeirão Preto, São Paulo, Brazil.

**Keywords:** Dental resorption, External cervical resorption, Dental trauma, Concussion

## Abstract

External cervical resorption is caused, almost exclusively, by dental trauma - especially those characterized by concussion - and is a dental disease to be diagnosed and treated accurately by endodontists. However, the vast majority of the cases is initially diagnosed by an orthodontist, due to the imaging possibilities in standardized documentations. Among the causes of external cervical resorption, it is common to mistakenly attribute it to orthodontic treatment, traumatic occlusion or even to chronic inflammatory periodontal disease. External cervical resorption is associated to dental trauma in several situations mentioned in this paper. In old cases, and eventually still nowadays, it may have been induced by internal tooth bleaching, which is increasingly less frequent in endodontically treated teeth. There are some tips to be followed and some care that must be taken during the diagnosis and treatment of external cervical resorption clinical cases. The present study lists foundations that will allow the professional to perform safely and accurately in each specific case. Some of these tips and care measures are of orthodontic nature.

## RESUMO

A reabsorção cervical externa é provocada, quase exclusivamente, pelo traumatismo dentário, especialmente os do tipo concussão. Ela constitui uma doença dentária a ser diagnosticada e tratada com precisão pelo endodontista, mas a grande maioria dos casos é inicialmente diagnosticada pelo ortodontista, em função das oportunidades imagiológicas nas documentações padronizadas. Entre as causas da reabsorção cervical externa, é comum atribuí-la equivocamente ao tratamento ortodôntico, à oclusão traumática ou, ainda, à doença periodontal inflamatória crônica. A reabsorção cervical externa está associada ao traumatismo dentário em várias situações mencionadas neste trabalho. Em casos mais antigos - e ainda hoje, eventualmente -, pode ter sido induzida pelo clareamento dentário interno, cada vez menos realizado em dentes endodonticamente tratados. Algumas dicas e cuidados devem ser tomados no diagnóstico e no tratamento dos casos clínicos de reabsorção cervical externa. No presente trabalho, são listados fundamentos para que o clínico possa atuar com segurança e precisão em cada caso, especificamente. Nessas dicas e cuidados, encontram-se alguns de natureza ortodôntica.

## Some dental clinicians often question whether dental resorptions have been diagnosed more than they used to be, or if their frequency has been increasing. 

Dental resorptions have been increasingly diagnosed in the clinical planning of all specialties due to the following:

1) People having more access to advanced means of diagnosis, that is, digital radiography and computed tomography with 3D reconstruction and slices in all directions and plans.
2) The higher level of knowledge of this issue by the professionals, which leads to identifying more cases at their early stages.
3) The fact that tooth loss related to dental caries and to chronic inflammatory periodontal disease associated to the dental bacterial plaque has decreased in the last decades.

Tooth preservation and the improvement of quality of life increase the chances of dental traumas resulting from leisure, sports, domestic or work activities. 

Dental trauma increasingly represents the major cause of tooth loss, as a result of the dental resorption that it induces in its several forms. 

Dental trauma may also lead to tooth loss due to dental and alveolar fractures, as in the avulsions in which the tooth was not found after the accident. 

Among dental resorptions, the one that is less taught and learned in formal graduation and specialization courses is the external cervical resorption, whose some important aspects will be dealt with in this paper in order to base diagnoses and treatment plans. 

## WHY DOES IT HAPPEN?

Although it occurs more frequently in upper anterior teeth, external cervical resorption may occur in any permanent teeth and it begins in the small windows or gaps of the cementoenamel junction dentine. These windows are protected by the gel of the normal gingival connective tissue represented by the extracellular matrix.

When this region is suddenly exposed to inflammation, by the dental trauma, without the junctional epithelium apical migration happening, the gel aforementioned will dissolute and the dentine will be exposed and recognized as antigenic by the immune system, if we consider that there are proteins in it that are not recognized by one's organism as belonging to it. 

**Figure 1:**
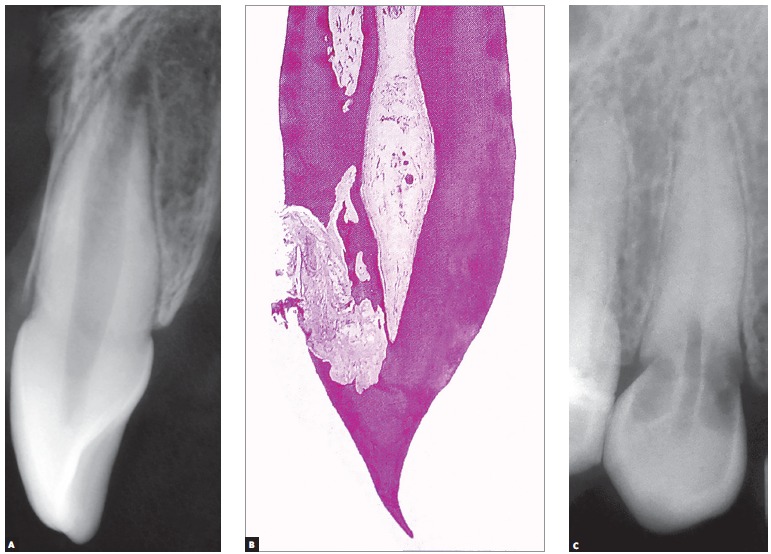
Mono-radicular tooth radiographically sound, for comparative purpose with B, in which the resorptive external cervical process reveals, microscopically, preservation of the pulpal limits, due to the fact that the non-mineralized predentine is not an ideal spot for fixing the clasts - which requires mineralized surfaces - preserving the periodontal space, as it can be seen in the radiography in C. The treatment is able to preserve the dental pulp, which remains normal, without inflammation or accelerated ageing.

The immune system in this area is represented by the macrophages, which are the cells that continuously runs or reside in basically all of our tissues. The way that the organism finds to remove these proteins is the action of the clasts over the dentine, resulting in the external cervical resorption (Fig 1). 

In the periodontal disease, slowness of the process leads the junctional epithelium to proliferate and migrate to apical, resurfacing and/or placing the cementoenamel junction on the gingival sulcus or on the periodontal pocket. Thus, the dentine cannot be recognized as foreign, as it will no longer be exposed to the gingival connective tissue. 

## HERE IS ITS CAUSE IN THE SEVERAL CLINICAL SITUATIONS!

**Figure 2:**
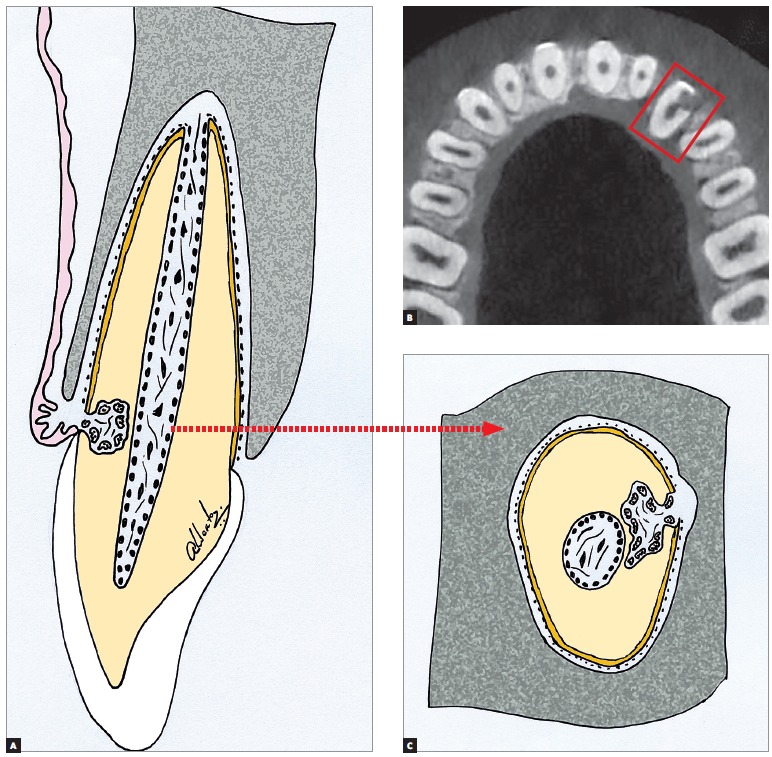
External cervical resorption schematically shown (A and C), from tomographic slices (B). The predentine and the remaining mineralized dentine are preserved, for the clasts do not get attached to non-mineralized tissues. The pulpal limits remain like that indefinitely over time, in spite of the extension of the resorptive process, which characterizes the most typical imaging signal of the process. For these reasons, the treatment is capable of preserving the dental pulp, which remains normal, not showing inflammation or accelerated ageing.

**Figure 3:**
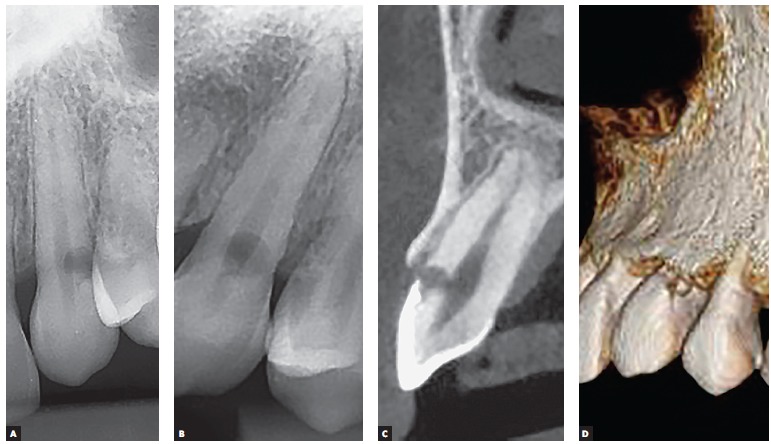
Upper canine with external cervical resorption, associated to transurgical dental trauma, in which the most characteristic radiographic signal stands out: the maintenance of the pulpal limits superimposed to the resorption area (A, B). In the tomography, the thinness of the predentine and of the preserved remaining tooth structure, do not result in observable image, even though it is always present in teeth with preserved pulp (C). In D, the 3D reconstruction highlights the area of the external cervical resorption where the process started and communicates with the gingival tissues. For these reasons, the treatment can preserve the dental pulp, which remains normal, not showing inflammation or accelerated ageing.

1) The cause currently involved in almost all the cases of external cervical resorption is dental trauma, mainly represented by a trauma of concussion type which the patient does not recall, omitting it during the anamnesis. Nevertheless, other types of dental trauma are associated with external cervical resorption.
2) Use of surgical levers that anchor on surrounding teeth to perform extractions.
3) Surgical procedures performed in surrounding teeth that lead to manipulation of their cervical region, as it may occur in procedures for implant placement.
4) Surgical manipulation of the cervical region of non-erupted canines for bonding brackets or devices aiming at orthodontic traction (Figs 2 and 3). Such manipulation is generally associated with the cervical removal of the pericoronal follicle, and shows to be unnecessary for the procedure itself. Whenever it is possible, one should preserve 1 to 2 millimeters of the pericoronal follicle adhered to the cervical region, which, for its turn, must be protected and preserved from surgical manipulations.
5) Surgical manipulation of the cervical region in the distal of the second molars during procedures for extraction of third molars.
6) A situation that used to be very common some decades ago and is related to the external cervical resorption is internal tooth bleaching. However, it has become increasingly less frequent.

The available options for an efficient bleaching that does not harm the dental tissues nor represent risks of external cervical resorption are almost a unanimous choice, even in those cases of discoloration of teeth endodontically treated. The efficient options that make use of facets and derived technologies contribute, to a great extent, to the low incidence of internal dental bleaching cases. 

On average, 10% of the cases of internally bleached teeth showed external cervical resorption as a consequence. The hydrogen peroxide liberated during the internal tooth bleaching presents wide permeability in the dentine and gets out through the dentine gaps in the amelodentinal junction of all human teeth. When it gets out through the dentinal tubules that open in the dentine gaps, its tissue toxicity induces inflammation in the adjacent connective and dissolution of the extracellular matrix. Without the extracellular matrix gel, the exposed dentine will be recognized as antigenic and then starts the process of external cervical resorption, as explained before. 

## TIPS AND CARE IN THE DIAGNOSIS AND TREATMENT PLAN OF EXTERNAL CERVICAL RESORPTION IN THE ORTHODONTIC CLINICAL PRACTICE

1) Informing the cause: the patient should be told that the cause of external cervical resorption in teeth without endodontic treatment must always be attributed to dental trauma, in its several forms, as previously mentioned.

The external cervical resorption cases in endodontically treated teeth, and that have no bleaching history, should also be attributed to dental trauma. 

Orthodontic treatment, traumatic occlusion and periodontal disease do not cause external cervical resorption. Orthodontic treatment and traumatic occlusion do not reduce the blood flow to the cervical region of the tooth, since gingiva is irrigated and shows vascular plexus fully nourished by various vessels that come from the buccal mucosa, the alveolar bone and the periodontal ligament. 

In the chronic inflammatory periodontal disease associated to the dental bacterial plaque, one of the first phenomena is the epithelial proliferation, with hyperplasia of the long junctional epithelium and its migration in apical direction, resurfacing the region of the cementoenamel junction with their dentine gaps, placing them under the epithelium and/or in the gingival sulcus and, then, in the periodontal pocket. 

2) Respecting the pupal limits: external cervical resorption, as any other external resorption, respects the pulpal limits due to the fact that the clasts do not get fixed to non-mineralized tissues, as the predentine (Figs 1, 2 and 3). This sign is determining in the differential diagnosis with the internal resorption. In radiographic images and in tomographic slices, the pulpal limits will be always preserved and the external resorption often circumvents the pulpal limits. 

3) The pulp is normal: another aspect that is cause of concern to all professionals refers to the pulpal impairment and the need of endodontic treatment, or not, in the treatment context. 

The remainder of the dentine between the external cervical resorption and the dental pulp is too thin and little mineralized, so it does not offer a radiographic image capable of delineating the pulpal limits in that region, conveying a false impression of impairment of the pulp space (Figs 1, 2 and 3).

4) It does not soften the dentine: external cervical resorption does not act deeply in the dentine. Rather, it does only in the interface between the clasts and the mineralized tissue, even if there are dentinal tubules. The enzymes and acids liberated by the clasts do not hit the pulp, even when it is very close. That is, even if the external cervical resorption is profound and extensive, it does not induce dental pulp inflammation or its necrosis - the pulp remains totally normal, in all aspects. 

5) Endodontics merely as a clinical convenience: according to the dimension of the external cervical resorption, its filling and the pulpal protection avoid the preservation of the pulpal limits and, as a surgical and clinical convenience, the endodontic treatment is required for the complete closure of the spaces of the coronary and radicular structures reabsorbed. This need can generally be evaluated and planned, but can be eventually detected during the surgical procedures. 

6) Not enlarging the gap, if possible! It is important to detect in the images the face on which the gap and the initial place of the process is. In general, the external cervical resorption would force down the surrounding tissues with a higher amplitude than its affected area on the radicular surface. 

7) As for vigorous curettage: do not! One of the questions refers to the need of curettage of all surfaces with resorption, even the deeper areas. Vigorous and unrestricted curettage of all areas is not necessary and should not be performed as it is in dentine caries, where there is the attempt of eliminating all the dentine softened by the microbial acids. Dental resorption does not previously soften the dentine, at any depth level. The acids and enzymes of the clasts act only on the surface between the clasts and the dentine, without invading the profound regions, as it happens in caries. 

In many cases, much more than curettage is needed. Instead, there should be, via a dental root resorption window, an irrigation with alkaline substances, such as water of calcium hydroxide, which will remove the clastic cells by means of the flow and high pH.

After these procedures and once the teeth surface is dry, it is possible to promote the pulpal protection with adequate material and by filling the spaces with material that, on the surface, shows to be as compatible as possible with the biology of the periodontal tissues, but especially the long junctional epithelium, which will recover the region and enable a more clinically normal anatomy and physiology, as much as possible. 

8) Irrigation: flow and pH: the irrigation of the area removes and/or disorganize the bone remodeling units and their clasts, as well as close their source of origin, as it comes from the gingival connective tissues, via superficial opening of the external cervical resorption. The filling of this area and the regularization of the surface cease the process and its recurrence is not detected. 

The use of substances with alkaline pH, as mentioned, must provide better conditions to access the resorption process.

9) Structure embrittlement: another important point to be highlighted in the external cervical resorption is the embrittlement of the dental structure. In more serious cases, where the cervical root is considerably involved, it is necessary to evaluate the convenience of conservative therapy, as fractures may occur secondarily in the brittle area and that risk should be evaluated at the planning stage. 

10) Gingival state: the gingiva will hardly present early warning signs of inflammation in the cervical region where the resorption window is opened, that is, where the process began. 

Initially, there will be an increase in volume and hyperemia in the region. In the more vigorous investigation of the gingival sulcus, an gap in the area where the process started is detected when the external cervical resorption is at an advanced stage. 

At the early stages, this opening cannot be spotted yet, as it is protected by the sulcular and the junctional epithelium, and is directly related to the connective gingival tissue.

11) Pink stained enamel: it may not be internal! In a few cases, the main patients' complaint refers to cervical pink stains on the crown. Tis pinkish area is generally considered typical of an internal coronary resorption. However, in some cases, it may actually be an external cervical resorption case, which moved towards the enamel, undermining the dentine and exposing the subjacent tissue.

Radiological images will differ the origin and the diagnosis of the process, that is, whether it is internal or external, which will redefine the treatment plan. If the resorption is internal, then the approach will be pulpal. On the other hand, in case of external cervical resorption, then the approach should be periodontal. 

## WHICH SPECIALTIES ARE INVOLVED?

All dental resorptions show prevalence of 5 to 10% in Western population and it is acknowledged in the Dentistry field that Endodontics is the specialty that studies, diagnoses and treats dental resorptions, except for those associated with the orthodontic movement. 

In Endodontics courses and training programs, the subject is recurrent and repeatedly dealt with, even though the majority of dental resorptions is carried out through the periodontal ligament. External cervical resorption, in the same way as lateral and apical external resorptions, demands an approach that involves an endodontist whenever operative interventions and/or establishment of prognosis are required. 

External cervical resorption should be dealt with by an endodontist who is prepared for paraendodontic surgeries and has esthetic and periodontal abilities simultaneously, in order to recover the good condition of the associated periodontal tissues, the resorbed surface and the pulpal protection. The professional must also have abilities related to imaging diagnosis and the interpretation of its nuances. All this background must be based on profound knowledge of pulpal and periodontal biopathology. 

When it comes to a patient who will undergo orthodontic treatment or in orthodontic treatments in which external cervical resorption has been diagnosed, the case must be dealt with by an endodontist who is able to coordinate a team in which there is a periodontist and a beautician, depending on how complex the case is. 

## CAN A TOOTH WITH TREATED EXTERNAL CERVICAL RESORPTION BE ORTHODONTICALLY MOVED? HOW TO PROCEED? 

Once the radicular surface and the good condition of the peripheral tissues are reestablished, the orthodontic treatment should start or continue. This period normally lasts around a month. This cervical region does not suffer circulatory disruptions nor inflammatory process resulting from the orthodontic movement. It should also be mentioned that the orthodontic forces, even the most intense ones, are spread all over the root. Furthermore, they show low magnitude and are dissipating. 

An important point is the meaning of external cervical resorption as a tooth that was subjected to dental trauma. In orthodontic practice, one of the predictive factors of the more severe dental resorptions during the orthodontic movement is the previous dental trauma, even if it appears to be a simple concussion. 

It fits the rule according to which previously traumatized teeth must be orthodontically moved in a different way. Teeth presenting external cervical resorption are previously traumatized teeth. The forces should be better distributed and imaging follow-up should be done every three months during the orthodontic treatment. 

## FINAL CONSIDERATIONS 

External cervical resorption is caused, almost exclusively, by dental trauma, especially concussion, and it is a dental disease to be accurately diagnosed and treated by an endodontist, although the vast majority of the cases is at first diagnosed by an orthodontist. 

Commonly, the cause of external cervical resorption is mistakenly attributed as if it were induced by orthodontic treatment, by traumatic occlusion or even chronic inflammatory periodontal disease. External cervical resorption is associated to dental trauma in many situations listed in this paper. In old clinical cases, it may eventually have been induced by internal tooth bleaching, which is increasingly less frequent when it comes to endodontically treated teeth. 

Some tips and care measures must be considered for the diagnosis and treatment of the clinical cases of external cervical resorption. This paper has presented the foundations to enable the professional to perform safely and accurately in each specific case. Some of these tips and care procedures are of orthodontic nature. 
